# The Genoa Vascular Biobank: A Today Resource for Future Perspectives in Vascular Research

**DOI:** 10.1177/11772719251324322

**Published:** 2025-07-13

**Authors:** Chiara Barisione, Jorge Miguel Mena Vera, Caterina Ivaldo, Silvia Ortona, Pier Francesco Ferrari, Paola Visconti, Michele Paudice, Martina Bastianon, Caterina Melani, Gaddiel Mozzetta, Valerio Vellone, Giovanni Pratesi, Domenico Palombo

**Affiliations:** 1 Department of Surgical and Integrated Diagnostic Sciences, University of Genoa, Genoa, Italy; 2 IRCCS Ospedale Policlinico San Martino, Genoa, Italy; 3 Research Center for Biologically Inspired Engineering in Vascular Medicine and Longevity, University of Genoa, Genoa, Italy; 4 Department of Experimental Medicine, University of Genoa, Genoa, Italy; 5 Department of Civil, Chemical and Environmental Engineering, University of Genoa, Genoa, Italy; 6 Department of Pathological Anatomy, IRCCS Ospedale Gaslini, Genoa, Italy

**Keywords:** Vascular surgery, abdominal aortic aneurysm, carotid artery stenosis, vascular biobank workflow

## Abstract

**Background:**

Biological knowledge and patient care have been significantly improved by the emergence of big data analysis and -omics sciences, requiring high quality standards for biospecimen and data collection. Biobanks are complex and dynamic units designated to fulfill these needs.

**Objectives:**

The Genoa Vascular Biobank (GTB-VD) is a collaborative network between the IRCCS Ospedale Policlinico San Martino (Centre of Biological Resources), and the University of Genoa (Vascular and Endovascular Surgery Unit; Anatomic Pathology Unit; Laboratory of Clinical and Experimental Vascular Biology). This work describes workflow, ethic and governance requirements, demographic and clinical characteristics of subjects enrolled in the GTB-VD, and the volume of open or endovascular surgical interventions.

**Design:**

The GTB-VD recruits patients undergoing surgical repair for carotid artery stenosis (CS) and abdominal aortic aneurysm (AAA), enrolled on the basis of selection criteria and subdivided for pathology and type of intervention, upon informed consent.

**Methods:**

Biospecimens comprise serum, plasma, whole blood, peripheral blood mononuclear cells, and urine (from AAA only), stored at −80°C; lesions from open surgeries are frozen and formalin fixed paraffin embedded. Samples are associated with donor’s clinical data through pseudonymization to prevent patient identification. Data accuracy and sample quality are ensured by harmonized standard operative procedures.

**Results:**

From 2018 to the end of 2023, 442 CS (distinguished into severe-asymptomatic or symptomatic, displaying a ratio of 5:1) and 214 AAA have been collected. CS is more frequently associated with diabetes and peripheral artery diseases, AAA with pulmonary history, and renal function impairment. Open surgery is more used for CS and endovascular for AAA.

**Conclusion:**

The GTB–VD, as organized, represents an “*unicum*” in our Country; it supports studies to identify molecular targets and biomarkers associated with specific arteriopathy, for developing secondary prevention strategies and minimally invasive, in situ therapies. Collaborative studies and sample sharing are welcome.

## Introduction

Over the past 2 decades, advancements in biological knowledge and patient care have been significantly enhanced by the emergence of -omics sciences (genomics, transcriptomics, proteomics, and metabolomics). To ensure the generation of comparable data and their integration with information for analysis, these techniques require standardization of both biospecimen quality and data collection,

To meet these needs, biobanks have evolved from simple biological sample repositories into complex and dynamic units integrated into infrastructure networks. Academic Medical Centers are ideal to manage biobanks because of their ability to provide access to human subjects, biological specimens, and clinical data. Furthermore, biobanking activities align with their mission of education, research, and patient care.^1^

Biobanks can be classified as “population-based,” containing epidemiological data collected from patients or volunteers without specific inclusion or exclusion criteria, or “disease-oriented,” focusing on specific populations with particular diseases, as frequently occurs for oncological and cardiovascular studies.

Our research unit (Vascular and Endovascular Surgery Unit) embarked on this journey approximately 15 years ago by collecting specimens and clinical data from patients suffering from different vascular diseases.^2-15^ Over time, this activity has fueled collaborative research projects with institutional, national, and international teams.

We are focused on patients addressed to surgery for carotid artery stenosis (CS) and abdominal aortic aneurysm (AAA), to prevent the potentially devastating consequences of ischemic stroke, due to carotid plaque embolization, and abdominal aortic rupture. To date, the risk stratification is calculated from parameters on arterial imaging,

The Genoa Vascular Biobank has enrolled CS and AAA patients since 2018: systematically, subject recruitment is based on inclusion and exclusion criteria, biological specimens are collected under controlled conditions, and personal data are organized as reported in this work.

This manuscript provides an overview of the network, workflow, ethical and legal considerations, and available resources of the Genoa Tissue Bank—Vascular Division (GTB-VD). The mission of the GTB-VD is to contribute to the identification of molecular targets and circulating biomarkers specifically associated with atherosclerotic complications, supporting advancement for risk stratification and personalized interventions in the field of artery diseases.

## Methods

### Network composition and workflow

The GTB-VD comprises the Vascular and Endovascular Surgery Unit, the Laboratory of Experimental and Clinical Vascular Biology (BioVasc Lab), and the Anatomic Pathology Unit within the Department of Surgical and Diagnostic Sciences at the University of Genoa. Storage facilities are managed by the Biological Resource Center at the Scientific Institutes of Hospitalization and Care (IRCCS) Ospedale Policlinico San Martino (CRB-HSM).

The 4-step workflow is:

1. Recruitment of Participants and Biospecimen Collection:

Patients admitted to the Vascular and Endovascular Surgery Unit for elective surgical correction of CS and AAA are selected 1 day before the intervention. If eligible for biobanking, they can be enrolled upon informed consent.

Criteria of inclusion: patients admitted to the Vascular and Endovascular Surgery Unit for open or endovascular surgery of both symptomatic and severe-asymptomatic CS and AAA.

Criteria of exclusion: serological positive for HIV, HBV, HCV; history of recent or ongoing neoplasia or anti-tumor therapy in the previous year; myocardial infarction in the 6 months before surgery; presence of autoimmune diseases.

The day before the intervention, peripheral blood is collected from all the participants, urine samples from AAA patients only; tissue samples are available when open surgery is performed.

The hospital transport team is responsible for delivering specimens, at room temperature, within 2 hours from the Vascular and Endovascular Surgery Unit to the BioVasc Lab.

2. Reception and Processing of Samples:

Upon sample arrival at the BioVasc Lab, operators assess its quality, including factors such as the elapsed time between collection and delivery and the presence of hemolysis in plasma or serum. An alphanumeric code is electronically assigned to each patient and recorded on labels for cryovials containing frozen specimens and jars with 10% formalin for tissue biopsies. Samples that do not meet the criteria for biobanking are disposed of after informing the patient, who is required to sign a specific form. Blood, urine, and tissue samples are processed in a dedicated room under a sterile laminar flow hood as described below in the “Biospecimen collection and processing” paragraph.

3. Storage:

Peripheral blood-derived and frozen tissue samples are stored in monitored and alarmed facilities at −80°C, managed by the CRB-HSM. Formalin-fixed, paraffin-embedded (FFPE) samples are stored in histotheques of the Anatomic Pathology Unit, in cardboard boxes at room temperature, protected from dust, light, and heat.

4. Data collection:

Clinical data collections are housed in the electronic database system of the Italian Society of Vascular and Endovascular Surgery (https://www.sicvereg.it/). Patients are registered with the study number electronically assigned to prevent subject identification.

### Ethics and governance of Genoa Tissue Bank -VD

The Vascular Bank Division (VD) is a part of the Genoa Tissue Bank (GTB) of the IRCCS Policlinico San Martino Hospital and belongs to the Institute’s Biological Resources Center (CRB-HSM).

The collection of biological samples for research purposes occurs only after obtaining written informed consent (authentic, free, autonomous, convinced, responsible) from the Participant, previously provided with adequate information (according to the Privacy Code and the Provisions of the Privacy Guarantor). Information leaflets and consent forms are published by the Institution (CONSAZHQA_0015_GB_Biobanche_Centro_Risorse_Biologiche_(CRB)_Informativa_ai_sensi_degli_artt._12_13_14_Regolamento_UE_2016_679_INGLESE.PDF).

Samples and data collected in the CRB are accessible to the scientific community through an access procedure compliant with the indications of the GDPR (REGULATION (EU) 2016/679 OF THE EUROPEAN PARLIAMENT AND OF THE COUNCIL, 27th April 2016, on the protection of natural persons concerning the processing of personal data, their free movement (repealing Directive 95/46/EC, General Data Protection Regulation - GDPR), and the general access conditions of the Biobanking and BioMolecular resources Research Infrastructure - European Research Infrastructure Consortium (BBMRI-ERIC), of which the CRB is part.

Samples and data are distributed according to the “equitable access” and “scientific excellence” principles. Projects must be approved by the institutional Research Ethics Committee (REC) and by the internal CRB-HSM Evaluation Committee.

### Pre-surgery evaluation

Patients undergo a preoperative evaluation that includes assessments such as blood tests, electrocardiograms, chest X-rays, cardiology consultations, and risk stratification. Depending on the patient’s comorbidities, additional examinations may be performed.

– *CS*: patients receive a duplex ultrasound evaluation to assess the degree of stenosis, hemodynamic impact, plaque characteristics, and presence of micro-emboli; computed tomography angiography (CTA) of the neck and intracranial vessels is performed to evaluate the aortic arch and carotid anatomy. In some cases, magnetic resonance imaging (MRI) can be used as second-level diagnostic imaging if CTA is contraindicated.^16^

The carotid plaque rupture with subsequent embolism of a locally formed thrombus or plaque debris causes stroke or transient ischemic attack, causing neurological symptoms.^17^

CS is defined as symptomatic (SCS) when the last congruent cerebral (hemiplegia, hemiparesis) or retinal ischemic episode (transient monocular blindness or amaurosis fugax, or retinal infarction) occurred in the patient within the previous 6 months. However, based on reviews, the experts’ panel suggests that CS should be defined as symptomatic when the last symptoms occurred within the previous 3 months.^18^

We distinguish SCS from severe asymptomatic CS (ACS) patients.

Stenosis severity is estimated following 2 criteria: in the European Carotid Surgery Trial (ECST), the denominator was the estimated vessel diameter where the residual luminal diameter was measured (usually the carotid bulb); in North American Symptomatic Carotid Endarterectomy Trial (NASCET), the denominator is the diameter of a disease-free ICA segment above the stenosis, where the vessel walls were parallel. Both used minimum residual luminal diameter as the numerator.^19^

**Figure 1.**
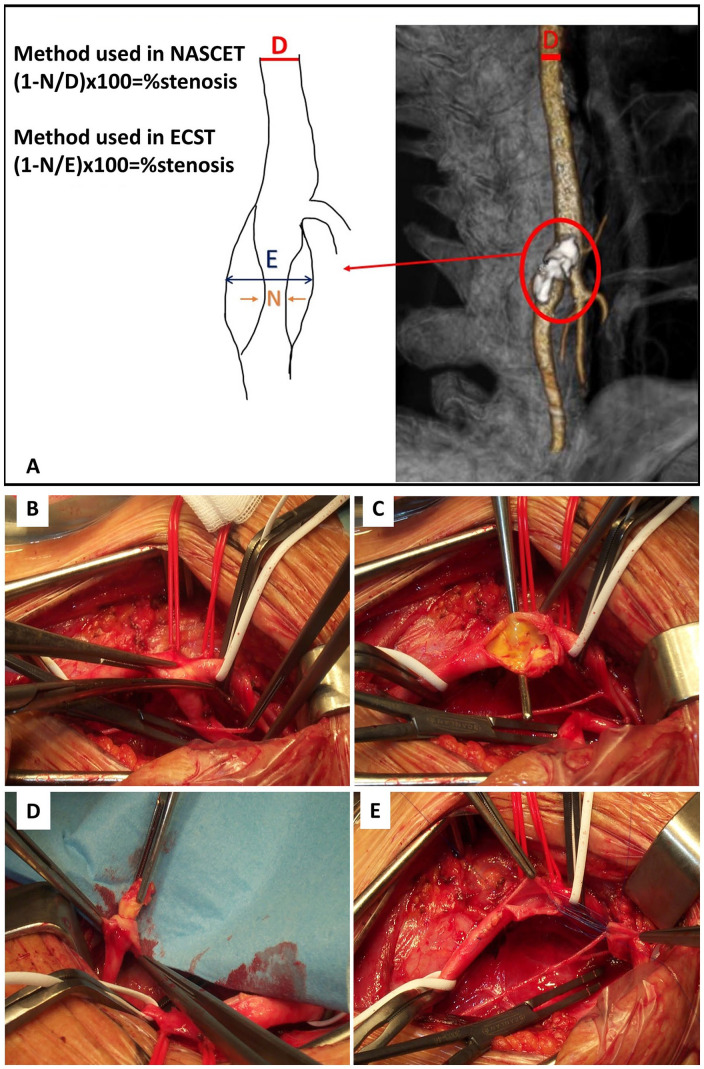
Schematic representation of carotid bifurcation and internal carotid artery, showing parameters to measure the degree of carotid stenosis on arterial angiography (A). Intraoperative images of carotid artery endarterectomy by eversion and reimplantation. (B-E): exposure of a carotid bifurcation with internal and external carotid artery (B); transected internal carotid artery: the vessel is straightened and the atherosclerotic plaque visualized. (C); Removal of the plaque by eversion. (D) transected internal carotid artery without the atherosclerotic plaque (E) Images are provided from our Unit of Vascular and Endovascular Surgery.

For ACS, current ESC guidelines put a threshold of 70% for formal indication. Revascularization should be discussed for SCS over 50% and for ACS over 60% (Figure 1A).

Surgical options for CS are Carotid EndoArterectomy (CEA), consisting of the removal of the atheroma, under local or general anesthetic, with no difference in the outcome (Figure 1B-E).^20^

Carotid Artery Stenting (CAS) is more frequently conducted under local anesthesia; it consists of the placement and expansion of the stent across the stenosis to restore the normal luminal diameter.^16^

Early trials reported a substantial reduction in stroke risk with carotid endarterectomy in patients with severe SCS, a modest benefit in patients with moderate symptomatic stenosis, as well as in patients with asymptomatic CS (Supplemental Table S1).^21^

– *AAA*: Elective surgery is indicated in case of a diameter exceeding 5 cm, fast growth, or peculiar morphological aspects indicative of increased risk of rupture. Options are Open Surgical Repair (OSR) or Endovascular Aortic Repair (EVAR), depending on the global evaluation of the patient (comorbidities, fragility, and aortic anatomy).

Diagnosis and prognosis of AAA rely on imaging techniques such as ultrasonography, the first-line imaging tool for detection and management of small AAAs^22,23^; CTA, providing a complete data set of the entire aorta, essential for EVAR^24^; MRI, has an advantage over CTA when AAA management requires repeated imaging,^25^ but is contraindicated in case of claustrophobia and metal implants; Positron Emission Tomography/Computed Tomography (PET-CT): provide clues on the presence of inflammatory cells.^24-27^

**Figure 2.**
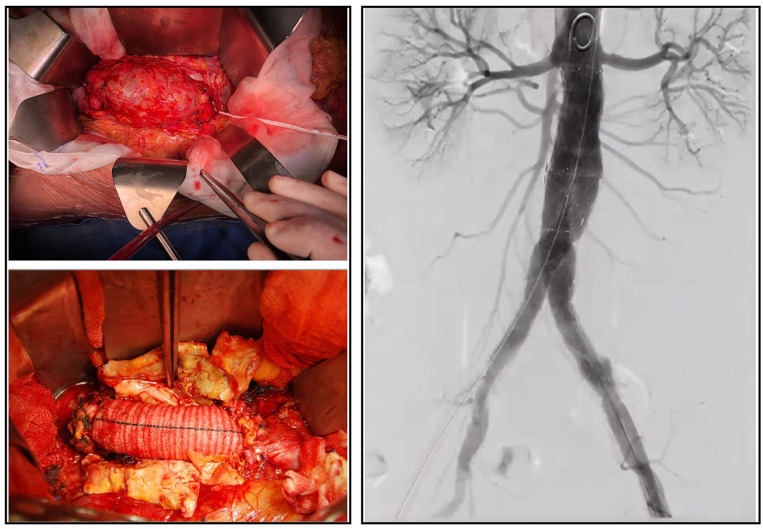
Open surgery: aorto-aortic graft for aneurysm repair (left panel); Intraoperative angiography of an endovascular aortic aneurysm repair (EVAR; right panel). Images are provided by our Unit of Vascular and Endovascular Surgery.

OSR is performed through an incision in the abdomen, replacing the AAA with a polyester (Dacron) graft. EVAR is carried out percutaneously: a stent is passed up from the arteries in the groin into the aorta, to seal the sac from the inside of the AAA (Figure 2).

### Biospecimen collection and processing

*Peripheral Blood - derived and urine samples*

Fasting peripheral blood is collected in 3 tubes, 6 mL of volume each (Vacuette Greiner Bio-One International GmbH), 1 containing a clot activator and gel separator for serum collection, the other 2 containing K3-EDTA for plasma, whole blood, and Peripheral Blood Mononuclear Cells (PBMCs) isolation.

Plasma and sera are processed by centrifugation at 3500 × *g* for 15 minutes at room temperature. After centrifugation, they are subdivided into 500 µL aliquots; whole blood into 250 µL aliquots; PBMCs are isolated from 6 mL of blood, as described in the Supplementary protocol (*Supplementary data -protocol*); urine samples are collected from AAA patients only, aliquoted in 6 vials (1 mL each). Aliquots are stored at −80°C.

#### Tissue sample

After surgical excision, tissue samples are transported in a dry, sterile, disposable container from the operating room to the BioVasc Lab. Two aliquots of approximately 0.5 to 1 g each are immediately stored at −80°C for molecular analysis.

**Figure 3.**
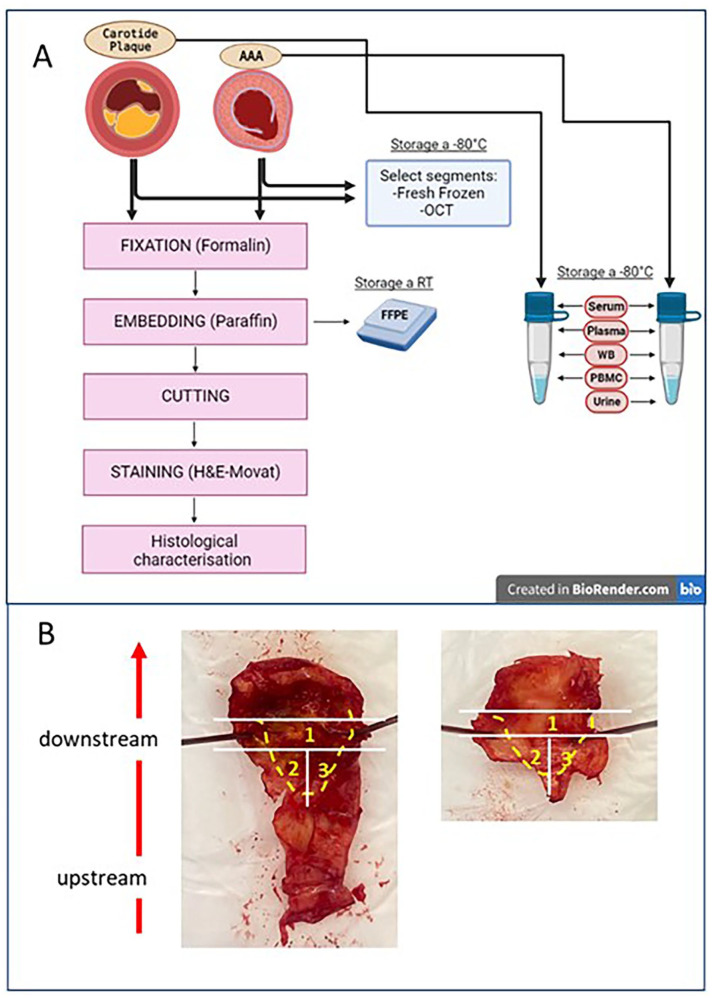
(A) Flowchart of vascular sample processing. Carotid plaque: lesion from patients with high/moderate-graded carotid symptomatic stenosis or with severe carotid asymptomatic stenosis; AAA: aortic wall from patients with abdominal aortic aneurysm FFPE: Formalin-Fixed Paraffin-Embedded; OCT: Optimal cutting temperature used to embed tissue samples prior to frozen sectioning on a microtome-cryostat; H&E: hematoxylin-eosin staining; Movat: Monat pentachrome staining; WB: Whole Blood; PBMCs: peripheral blood mononuclear cell. (B) Criteria for carotid plaque dissection and storage. When recognizable, the lesion is oriented according to the bloodstream: section 1, comprehending the core (in the middle) and the shoulder (at both sides), FFPE; sections 2 and 3, comprehending mostly plaque shoulder an, the core of the upstream portion of the plaque, are stored at −80°C. Often, the arterial wall (left side) and the inner side containing the plaque (right side) are divided into complementary fragments.

The remaining segments are fixed in 10% formalin at room temperature and then sent to the Anatomy Pathology Unit, for paraffin embedding according to standard protocols. FFPE specimens are then cut in slices (about 3 µm thick), stained with hematoxylin/eosin (H&E), and, on-demand, with Movat’s Pentachrome staining (MOVAT; Figure 3A).

Biopsies from CS comprehend the plaque, with core and shoulder, and a portion of the medial layer retrieved with the lesion. Considering this composition, aliquots are picked up as represented in Figure 3B.

Biopsies from AAA are often more abundant; therefore, all fragments represent the histology of the same lesion.

*Quality control tests on biological specimens*

When required, the RNA integrity number of dry-frozen PBMCs is assessed using the Agilent 2100 Bioanalyzer in combination with the RNA 6000 Nano Kit (Agilent Technologies, Waldbronn, Germany). This assessment is carried out in accordance with the manufacturer’s instructions.

FFPE section are then stained with H&E and MOVAT to evaluate tissue morphology.

## Results

### Study populations

Since November 2018, the GTB-VD has been accumulating biological samples from 656 subjects patients who have undergone surgery for CS and AAA (respectively CAS = 60; CEA = 382; EVAR = 132; OSR = 82).

Table 1 presents an overview of these demographic and clinical attributes to the pathology, further depicted in Table 2 as divided by type of surgery.

**Table 1.** Demographic and clinical characteristics of enrolled subjects. eGFR, estimated glomerular filtration rate; CKD, Chronic Kidney Disease; All data are presented as the median [IQR] or as mean (SD) depending on the result of normality testing.

Variable	AAA	Carotid.stenosis	*P* value
N	214	442	
Males (%)	189 (88.3)	303 (68.6)	*<*.001
Age (median [IQR])	77.00 [71.00, 82.00]	76.00 [70.00, 81.00]	.114
Pathology = AAA/Carotid stenosis (%)	214/0 (100.0/0.0)	0/442 (0.0/100.0)	*<*.001
Type of stenosis = sintomatica (%)	0 (0.0)	95 (21.5)	1.000
Diabetes (%)	23 (10.7)	107 (24.2)	*<*.001
Pulmonary history (%)	34 (15.9)	27 (6.1)	*<*.001
Cardiac history (%)	36 (16.8)	72 (16.3)	.952
Dyslipidemia (%)	77 (36.0)	190 (43.0)	.104
Hypertension history (%)	131 (61.2)	275 (62.2)	.871
Chronic kidney insufficiency (%)	28 (13.1)	44 (10.0)	.285
Peripheral artery disease(%)	5 (2.3)	38 (8.6)	.004
Leukocytes ×10E9/L (median [IQR])	6.90 [5.97, 8.24]	7.46 [6.29, 8.76]	.007
Erythrocytes ×10E12/L (median [IQR])	4.60 [4.20, 4.90]	4.40 [4.00, 4.80]	.002
Hemoglobin g/L (median [IQR])	139.00 [127.25, 149.00]	130.00 [119.00, 142.00]	*<*.001
Platelets ×10E9/L (median [IQR])	193.00 [165.00, 233.00]	215.00 [178.00, 261.00]	*<*.001
Neutrophils ×10E9/L (median [IQR])	4.52 [3.76, 5.42]	4.70 [3.83, 5.92]	.129
Lymphocytes ×10E9/L (median [IQR])	1.49 [1.18, 1.88]	1.67 [1.29, 2.10]	.009
Creatinine mg/dL (median [IQR])	1.00 [0.90, 1.20]	1.00 [0.80, 1.20]	.155
eGFR mL/m^2^/1.73mq (median [IQR])	70.50 [55.00, 84.00]	74.00 [52.00, 86.00]	.689
Stage 1 of CKD (%)	24 (11.2)	58 (13.1)	.571
Stage 2 of CKD (%)	104 (48.6)	170 (38.5)	.017
Stage 3a of CKD (%)	43 (20.1)	53 (12.0)	.008
Stage 3b of CKD (%)	11 (5.1)	46 (10.4)	.036
Stage 4 of CKD (%)	10 (4.7)	7 (1.6)	.038
Stage 5 of CKD (%)	0 (0.0)	5 (1.1)	.279

**Table 2.** Patient demographics and comorbidities of enrolled subjects, as divided for the type of surgery; eGFR, estimated glomerular filtration rate; CKD, Chronic Kidney Disease. All data are presented as the median [IQR] or as mean (SD) depending on the result of normality testing.

Variable	CAS	CEA	*P* value
N	60	382	
Males (%)	45 (75.0)	258 (67.5)	.314
Age (median [IQR])	76.50 [70.75, 80.25]	76.00 [70.00, 81.00]	1.000
Type of stenosis = sintomatica (%)	12 (20.0)	83 (21.7)	.894
Diabetes(%)	14 (23.3)	93 (24.3)	.994
Pulmonary history (%)	5 (8.3)	22 (5.8)	.628
Cardiac history (%)	14 (23.3)	58 (15.2)	.161
Dyslipidemia(%)	21 (35.0)	169 (44.2)	.229
Hypertension history (%)	34 (56.7)	241 (63.1)	.418
Chronic kidney insufficiency (%)	5 (8.3)	39 (10.2)	.826
Peripheral artery disease(%)	2 (3.3)	36 (9.4)	.188
Leukocytes ×10E9/L (median [IQR])	7.44 [6.35, 8.60]	7.47 [6.25, 8.80]	.799
Erythrocytes ×10E12/L (median [IQR])	4.50 [4.00, 4.80]	4.40 [4.00, 4.80]	.675
Hemoglobin g/L (median [IQR])	130.00 [118.00, 138.00]	130.00 [119.00, 143.00]	.603
Platelets ×10E9/L (median [IQR])	218.00 [183.00, 261.00]	214.00 [178.00, 259.50]	.474
Neutrophils ×10E9/L (median [IQR])	4.77 [3.89, 5.88]	4.70 [3.80, 5.93]	.776
Lymphocytes ×10E9/L (median [IQR])	1.66 [1.50, 2.07]	1.67 [1.29, 2.10]	.989
Creatinine mg/dL (median [IQR])	1.00 [0.90, 1.15]	1.00 [0.80, 1.30]	.627
eGFR mL/m^2^/1.73mq (median [IQR])	70.00 [59.00, 84.00]	74.00 [50.75, 86.00]	.837
Stage 1 of CKD (%)	7 (11.7)	51 (13.4)	.878
Stage 2 of CKD (%)	34 (56.7)	136 (35.6)	.003
Stage 3a of CKD (%)	6 (10.0)	47 (12.3)	.767
Stage 3b of CKD (%)	7 (11.7)	39 (10.2)	.907
Stage 4 of CKD (%)	1 (1.7)	6 (1.6)	1.000
Stage 5 of CKD (%)	0 (0.0)	5 (1.3)	.814
Variable	EVAR	OSR	*P* value
N	132	82	
Males (%)	118 (89.4)	71 (86.6)	.687
Age (median [IQR])	79.00 [73.00, 83.25]	74.50 [68.00, 79.00]	<.001
Diabetes (%)	15 (11.4)	8 (9.8)	.887
Pulmonary history (%)	21 (15.9)	13 (15.9)	1.000
Cardiac history (%)	26 (19.7)	10 (12.2)	.216
Dyslipidemia (%)	38 (28.8)	39 (47.6)	.008
Hypertension history (%)	81 (61.4)	50 (61.0)	1.000
Chronic kidney insufficiency (%)	13 (9.8)	15 (18.3)	.116
Peripheral artery disease(%)	3 (2.3)	2 (2.4)	1.000
Leukocytes ×10E9/L (median [IQR])	6.77 [5.72, 8.15]	7.13 [6.37, 8.50]	.132
Erythrocytes ×10E12/L (median [IQR])	4.50 [4.10, 4.90]	4.70 [4.40, 4.95]	.024
Hemoglobin g/L (median [IQR])	134.50 [124.00, 147.00]	144.00 [133.00, 149.75]	.001
Platelets ×10E9/L (median [IQR])	190.00 [162.75, 226.50]	207.00 [179.00, 239.00]	.084
Neutrophils ×10E9/L (median [IQR])	4.42 [3.79, 5.46]	4.65 [3.68, 5.39]	.761
Lymphocytes ×10E9/L (median [IQR])	1.41 [1.14, 1.75]	1.58 [1.26, 1.93]	.113
Creatinine mg/dL (median [IQR])	1.00 [0.90, 1.20]	1.00 [0.83, 1.28]	.899
eGFR mL/m^2^/1.73mq (median [IQR])	70.00 [53.50, 82.00]	73.50 [55.25, 87.00]	.323
Stage 1 of CKD (%)	11 (8.3)	13 (15.9)	.141
Stage 2 of CKD (%)	71 (53.8)	33 (40.2)	.074
Stage 3a of CKD (%)	25 (18.9)	18 (22.0)	.720
Stage 3b of CKD (%)	9 (6.8)	2 (2.4)	.275
Stage 4 of CKD (%)	6 (4.5)	4 (4.9)	1.000
Stage 5 of CKD = 0 (%)	0100.0)	0 (100.0)	NA

About 69% of CS and 89% of AAA patients are males. The prevalence of diabetes and peripheral artery diseases are significantly higher in CS patients, while pulmonary history (BPCO, emphysema) and renal function impairment, are more frequent among AAA subjects.

The more frequently applied techniques are CEA for CS and EVAR for AAA; while subgroups of CS patients do not significantly differ for the recorded clinical data, AAA patients that undergone EVAR are usually older and with lower hemoglobin levels in respect to those treated with OSR.

Our biobank encompasses a broad spectrum of ages, most subjects being over sixties. Tables 3 and 4 show the distribution by gender, age, and by type of intervention of CS, symptomatic and asymptomatic, and AAA, respectively. Of note, AAA repair occurs by OSR mainly among subjects aged 61 to 70 years, while by EVAR in older patients (81-90 years)

**Table 3.** Distribution by gender, age and by type of intervention of CS, symptomatic and asymptomatic.

Male CAS	asympto	sympto	*P* value	Male CEA	asympto	sympto	*P* value
Age	N = 35	N = 10		Age	N = 198	N = 60	
⩽50(%)	0 (100.0)	0 (100.0)	NA	⩽50(%)	0 (100.0)	0 (100.0)	NA
51-60(%)	1 (2.9)	3 (30.0)	.042	51-60(%)	10 (5.1)	3 (5.0)	1.000
61-70(%)	4 (11.4)	3 (30.0)	.350	61-70(%)	45 (22.7)	11 (18.3)	.586
71-80(%)	18 (51.4)	3 (30.0)	.402	71-80(%)	79 (39.9)	34 (56.7)	.032
81-90(%)	12 (34.3)	1 (10.0)	.272	81-90(%)	64 (32.3)	12 (20.0)	.094
⩾91(%)	0 (100.0)	0 (100.0)	NA	⩾91(%)	0 (100.0)	0 (100.0)	NA
Female CAS	asympto	sympto	*P* value	Female CEA	asympto	sympto	*P* value
Age	N = 13	N = 2		Age	N = 101	N = 23	
⩽50 (%)	0 (100.0)	0 (100.0)	NA	⩽50 (%)	0 (3.0)	0 (0.0)	NA
51-60 (%)	1 (7.7)	0 (0.0)	1.000	51-60 (%)	4 (4.0)	2 (8.7)	.677
61-70 (%)	3 (23.1)	0 (0.0)	1.000	61-70 (%)	17 (16.8)	5 (21.7)	.800
71-80 (%)	7 (53.8)	2 (100.0)	.642	71-80 (%)	52 (51.5)	7 (30.4)	.111
81-90 (%)	2 (15.4)	0 (0.0)	1.000	81-90 (%)	23 (22.8)	9 (39.1)	.176
⩾91 (%)	0 (100.0)	0 (100.0)	NA	⩾91 (%)	0 (2.0)	0 (0.0)	NA

**Table 4.** Distribution by gender, age and by type of intervention of AAA.

Male	EVAR	OSR	*P* value
Age	N = 118	N = 71	
⩽50 (%)	0 (0.0)	0 (0)	NA
51-60 (%)	2 (1.7)	3 (4.2)	.561
61-70 (%)	17 (14.4)	20 (28.2)	.034
71-80 (%)	49 (41.5)	38 (53.5)	.147
81-90 (%)	44 (37.3)	9 (12.7)	.001
⩾91 (%)	0 (5.1)	0 (0.0)	.133
Female	EVAR	OSR	*P* value
n	N = 14	N = 11	
⩽50 (%)	0 (100.0)	0 (100.0)	NA
51-60 (%)	14 (100.0)	11 (100.0)	NA
61-70 (%)	2 (14.3)	2 (18.2)	1.000
71-80 (%)	4 (28.6)	5 (45.5)	.650
81-90 (%)	8 (57.1)	4 (36.4)	.529
⩾91 (%)	0 (100.0)	0 (100.0)	NA

With the advent of endovascular techniques, the landscape of vascular surgery has witnessed a shift toward minimally invasive procedures. As represented in Supplemental Figure S1 (Supplemental Data), the plot illustrates the volumes of CS and AAA interventions/year since 2015 at our Unit, highlighting how endovascular interventions for AAA have surged in number. The record of this dynamic interplay between open and endovascular techniques enables their retrospective, long-term evaluation.

### Potential application for biomarkers evaluation of GTB-VD resources

The above data are reported anonymously and in an aggregated manner, enabling an overview of the available resources; based on the specific requirements, it is possible to choose and stratify cases with specific clinical pictures and laboratory parameters among those reported. This may provide useful insights to better evaluate the potential predictive value of the tested molecules, by identifying a set of potential biomarkers that, when combined, may increase their discriminative performance.

As reported in paragraph 2.1, patients are enrolled at a late stage of the disease, yet through tight criteria of exclusion, in order to avoid any biases related to chronic major comorbidities and treatments.

Therefore, the GTB-VD resources offer a trustable set of information for cross-sectional studies on advanced CS and AAA, paving the ground for future longitudinal studies and follow-up of the early stages of the diseases.

## Discussion

### Biobanks purposes and features

The harmonization and standardization of biobanks,^28^ have assumed paramount importance in the era of -omics sciences and systems medicine, marking a shift from reactive personalized care, which relies on responses to observed failures, to a precision-based approach.

According to the European Commission Joint Research Centre, modern biobanks exhibit key characteristics, including the collection and storage of biological materials linked to medical and epidemiological data, facilitating dynamic, continuous, and long-term collection.^29^ Moreover, they integrate with ongoing research, prioritize donor privacy through pseudonymization, and uphold governance standards for the future use of samples in diverse research projects.^30^

Several circumstances occurring before biospecimen collection can affect the reliability of analytical results. Variables related to subjects’ conditions (genotype, lifestyle, nutrition, medication, concomitant diseases, surgical interventions, etc.) cannot be standardized and deserve to be recorded in the associated database. The collection phase of biological material begins when the biobank’s staff receives samples. From then on, the state of the samples is influenced by transportation (compliance with the cold chain regime, duration), chain of custody in the laboratory, and procedures for isolating fractions for subsequent research. Finally, the proper record of samples enables the matching of information. These critical points can influence the total outcome of studies.^31^

Human biological samples need standardized practices for pre-analytical steps: blood requires careful consideration of anticoagulants to minimize biases in data collection, fresh or frozen tissues often yield higher-quality DNA and RNA, and technologies have adapted to test FFPE samples at room temperature.^31^

The publication of ISO 20387:2018 “Biobanking—General requirements for Biobanking” provides essential guidelines for the organization and processing of biological samples, ensuring minimum standardization requirements for the reproducibility and comparability of scientific research results. Ethical considerations, such as informed consent, sample ownership, confidentiality protection, veto rights, and biobank sustainability, are also addressed.^32,33^

### GTB-VD: our experience

Consistently with these main features, our experience of biobanking started from isolated studies on atherosclerosis, with a prominent focus on CS and AAA.

Atherosclerosis is a complex, systemic, and multifaceted disease. Borrowing an assumption from the experience of acute coronary syndrome,^34^ the complexity of atherosclerotic major events, as occurs also for ischemic stroke and abdominal aortic rupture, surpasses the traditional clinical categorization; so far based on ultrasound/CT image analysis oversimplifies a spectrum of possible manifestations and obscures mechanisms that may require different strategies of protection. Furthermore, this statistics-based criterion exposes to surgical procedures even subjects who would never encounter complications; on other side, it does not permit an early detection of those with a fast and ominous progression. Both CS and AAA still lack biomarkers to formulate precise prognoses and therapeutic molecular targets to reverse or just counteract the disease progression.

In the last 15 years, our research efforts in CS have unveiled crucial insights, such as the association between high serum lipoprotein(a) levels and acute coronary syndrome in severe CS patients.^9^

A milestone in the study of CS was added in 2010, with the observation that systemic and intraplaque inflammation, together, could influence global patient vulnerability for ischemic stroke; importantly, it has been pointed out that “orientation matters,” as the upstream or downstream portions of the plaque differently contribute to its vulnerability.^35^ Further observations recognized a protective role for Vitamin D receptor as an active anti-inflammatory mediator within atherosclerotic plaques,^36^ a predictive value of serum and intraplaque c-reactive protein (CRP) in patients with severe CS for major cardiovascular events,^10^ and an immunoregulatory role of resistin in inhibiting neutrophil-mediated atherosclerotic activities within the plaque.^11^ Lately, it has been shown that also ficolins may have a predictive value of cardiovascular disease symptoms, as they correlate with local and systemic inflammation, ultimately leading to plaque vulnerability.^12^

AAA is an aortic dilation potentially leading to vessel rupture, affecting mostly male subjects, smokers, or previous smokers, over 60.^37^ The Screening Abdominal Aortic Aneurysm Genoa (S.A.Ge) study showed the importance of an early screening program for AAA in 65- to 75-year-old men like a cost-effective strategy.^38^ The diagnosis through different imaging modalities assessing aneurysm size became quite relevant.

We previously observed that AAA is often associated with anemia, mild-to-moderate renal failure and cardiorenal syndrome.^6^ This finding prompted us to collect also urines from AAA patients to assess biomarkers of renal functions, providing suggestions for a more adequate type of intervention and imaging, that is, adopting CO_2_ for angiography instead of nephrotoxic contrast agents.

Since 2018, when the GTB-VD has been established, more than 656 cases have been recruited; of them, 34.6% underwent surgery for AAA repair and 65.4% for CS.

Looking at the surgical technique in AAA repair, 132 out of 214 cases are EVAR, indicating that, over time, the endovascular approach has become the most adopted, based on the need of the population. Our Unit represents a highly specialized center (regional hub for complex aortic surgery) of the University Hospital Policlinico San Martino IRCCS, an Academic Medical Center that provides health assistance for the population of Genoa and neighboring areas, characterized by the increased older classes compared to the national average (12.7% vs 11.1% in the 65-74 years old age group and 15.8% vs 11.7% for over 75, respectively; https://www.istat.it/it/file//2020/05/07-Liguria-Scheda).

## Conclusion

As a disease-oriented biobank, GTB-VD enables the comparison of cases according to different approaches within the vascular-diseased population, stratifying them on the basis of specific features such as symptoms, presence of comorbidity, etc. Adhesion to standard criteria of procedures and data collection also ensures the proper use of samples from different biobanks within the same project.

Currently, GTB-VD fuels projects to identify mechanisms, molecular targets, and biomarkers for optimizing new strategies of risk stratification and therapeutic options in AAA and CS respectively.

To this purpose, the contribution of GTB-VD in bioengineering research is mandatory: the identification of biomarkers is needed to design targeted approaches such as nanosystems and biosensors; vascular tissues and cells could serve for tissue engineering and regenerative medicine.

In the context of vascular drug delivery, our experience relies on hemocompatibility studies by using blood provided by the GTB to prove the hemocompatibility of nanoliposomes^39^ and antibody-decorated polymeric nanoparticles.^40^

The GTB-VD, as a member of the CRB-HSM, participates to the biobank network of the non-profit Association for the Study of Cardiovascular Diseases “Rete Cardiologica.” This latter streamlines biobanking activities across 20 IRCCS in Italy, adhering to shared procedures and ethical guidelines outlined in ISO 20387, with the goal of developing high-consistency research programs in cardiovascular diseases. Thus, we welcome collaborative proposals and sample contributions to our biobank, recognizing their pivotal role in advancing medical knowledge and improving patient care.

## List of Abbreviations

AAA: abdominal aortic aneurysm

ACS: Asymptomatic Carotid stenosis

BBMRI-ERIC: Biobanking and BioMolecular resources Research Infrastructure - European Research Infrastructure Consortium

BioVasc Lab: Laboratory of Experimental and Clinical Vascular Biology

CAS: Carotid Artery Stenting

CEA: Carotid EndoArterectomy

CRB-HSM: Biological Resource Center at the IRCCS Ospedale Policlinico San Martino

CRP: c-reactive protein

CS: carotid artery stenosis

CTA: computed tomography angiography

ECS: European Carotid Surgery

ECST: European Carotid Surgery Trial

EVAR: Endovascular Aortic Repair

FFPE: Formalin-fixed, paraffin-embedded

GDPR: General Data Protection Regulation

GTB-VD: Genoa Tissue Bank – Vascular Division

H&E: hematoxylin/eosin

IRCCS: Scientific Institutes of Hospitalization and Care

MOVAT: Movat’s Pentachrome staining

MRI: magnetic resonance imaging

NASCET: North American Symptomatic Carotid Endarterectomy Trial

OSR: Open Surgical Repair

PBMCs: Peripheral Blood Mononuclear Cells

PET-CT: Positron Emission Tomography/Computed Tomography

REC: Research Ethics Committee

S.A.Ge: Screening Abdominal Aortic Aneurysm Genoa

SCS: Symptomatic Carotid stenosis

VD: Vascular Bank Division

## Supplemental Material

sj-docx-1-bmi-10.1177_11772719251324322 – Supplemental material for The Genoa Vascular Biobank: A Today Resource for Future Perspectives in Vascular Research Supplemental material, sj-docx-1-bmi-10.1177_11772719251324322 for The Genoa Vascular Biobank: A Today Resource for Future Perspectives in Vascular Research by Chiara Barisione, Jorge Miguel Mena Vera, Caterina Ivaldo, Silvia Ortona, Pier Francesco Ferrari, Paola Visconti, Michele Paudice, Martina Bastianon, Caterina Melani, Gaddiel Mozzetta, Valerio Vellone, Giovanni Pratesi and Domenico Palombo in Biomarker Insights

sj-pptx-2-bmi-10.1177_11772719251324322 – Supplemental material for The Genoa Vascular Biobank: A Today Resource for Future Perspectives in Vascular Research Supplemental material, sj-pptx-2-bmi-10.1177_11772719251324322 for The Genoa Vascular Biobank: A Today Resource for Future Perspectives in Vascular Research by Chiara Barisione, Jorge Miguel Mena Vera, Caterina Ivaldo, Silvia Ortona, Pier Francesco Ferrari, Paola Visconti, Michele Paudice, Martina Bastianon, Caterina Melani, Gaddiel Mozzetta, Valerio Vellone, Giovanni Pratesi and Domenico Palombo in Biomarker Insights

## Declarations

**Ethics approval and consent to participate:** The GTB – VD is coordinated by the Biological Resource Centre (CRB-HSM), established in 2008 and recognized by Regione Liguria (DGR n. 34 22nd January 2010 and subsequent updates, DGR n. 34 of 01.22.2010, Ligurian Health Agency Resolutions n. 221 of 09.05.2018 and n. 307 of 08.27.2020). CRB-HSM is part of BBMRI.it, the National Node of the pan-European BioBanking and BioMolecular Resources Research Infrastructure (BBMRI-ERIC). In March 2010, the CRB obtained a favorable opinion from the Regional Ethics Committee for the use of Code of Ethics, Informed Consent to the storage of biological material for scientific research purposes, Confidentiality Agreement, Material Transfer Agreement. These documents and the CRB Regulation are shared with the GTB-VD and received a renewed favorable opinion from the Regional Ethics Committee of Liguria (approved meeting minutes 4/2021), upon deployment of the General Data Protection Regulation (EU) 2016/679 (GDPR).

**Consent for publication:** All patients’ images and data included have been obtained following the informed consent process. Upon admission to the ward, each subject is required to sign a consent form authorizing the use his/her clinical data and images for research and publication purposes. Therefore, patients are fully aware that their anonymized data and images may be included in subsequent scientific communications. All images have been processed to safeguard patient anonymity and confidentiality in accordance with institutional ethical guidelines and applicable data protection regulations.

**Availability of data and materials:** For details on data availability and sample sharing please refer to CRB-HSM Regulation.

**Agreement to submission:** All authors agree to submit the manuscript to Biomarker Insights

## References

[1] Montecucco F Carbone F Dini FL , et al. Implementation strategies of Systems Medicine in clinical research and home care for cardiovascular disease patients. Eur J Intern Med. 2014;25:785-794. 25283057 10.1016/j.ejim.2014.09.015

[2] Palmieri D Cafueri G Mongelli F , et al. Telomere shortening and increased oxidative stress are restricted to venous tissue in patients with varicose veins: a merely local disease? Vasc Med. 2014;19:125-130. 24557807 10.1177/1358863X14525002

[bibr3-11772719251324322] Cafueri G Parodi F Pistorio A , et al. Endothelial and smooth muscle cells from abdominal aortic aneurysm have increased oxidative stress and telomere attrition. PLoS One. 2012;7:e35312. 10.1371/journal.pone.0035312 PMC3325957 22514726

[bibr4-11772719251324322] Ghigliotti G Barisione C Garibaldi S , et al. CD16^+^ monocyte subsets are increased in large abdominal aortic aneurysms and are differentially related with circulating and cell-associated biochemical and inflammatory biomarkers. Dis Markers. 2013;34:131-142. 23348634 10.3233/DMA-120956 PMC3809748

[bibr5-11772719251324322] Barisione C Garibaldi S Furfaro AL , et al. Moderate increase of indoxyl sulfate promotes monocyte transition into profibrotic macrophages. PLoS One. 2016;11:e0149276. 10.1371/journal.pone.0149276 PMC4771744 26925780

[bibr6-11772719251324322] Barisione C Garibaldi S Brunelli C , et al. Prevalent cardiac, renal and cardiorenal damage in patients with advanced abdominal aortic aneurysms. Intern Emerg Med. 2016;11:205-212. 26510876 10.1007/s11739-015-1328-z

[bibr7-11772719251324322] Marini C Morbelli S Armonino R , et al. Direct relationship between cell density and FDG uptake in asymptomatic aortic aneurysm close to surgical threshold: an in vivo and in vitro study. Eur J Nucl Med Mol Imaging. 2012;39:91-101. 22012546 10.1007/s00259-011-1955-1

[bibr8-11772719251324322] Liberale L Bertolotto M Carbone F , et al. Resistin exerts a beneficial role in atherosclerotic plaque inflammation by inhibiting neutrophil migration. Int J Cardiol. 2018;272:13-19. 30075966 10.1016/j.ijcard.2018.07.112

[bibr9-11772719251324322] Pagano S Carbone F Burger F , et al. Anti-apolipoprotein A-1 auto-antibodies as active modulators of atherothrombosis. Thromb Haemost. 2016;116:554-564. 27356567 10.1160/TH16-03-0229

[bibr10-11772719251324322] Bonaventura A Mach F Roth A , et al. Intraplaque expression of C-reactive protein predicts cardiovascular events in patients with severe atherosclerotic carotid artery stenosis. Mediators Inflamm. 2016;2016:9153673. 27738391 10.1155/2016/9153673 PMC5050375

[bibr11-11772719251324322] Liberale L Carbone F Bertolotto M , et al. Serum adiponectin levels predict acute coronary syndrome (ACS) in patients with severe carotid stenosis. Vasc Pharmacol. 2018;102:37-43. 10.1016/j.vph.2017.12.066 29305337

[bibr12-11772719251324322] Carbone F Valente A Perego C , et al. Ficolin-2 serum levels predict the occurrence of acute coronary syndrome in patients with severe carotid artery stenosis. Pharmacol Res. 2021;166:105462. 33513354 10.1016/j.phrs.2021.105462

[bibr13-11772719251324322] Montecucco F Di Marzo V da Silva RF , et al. The activation of the cannabinoid receptor type 2 reduces neutrophilic protease-mediated vulnerability in atherosclerotic plaques. Eur Heart J. 2012;33:846-856. 22112961 10.1093/eurheartj/ehr449 PMC3345556

[bibr14-11772719251324322] Lenglet S Quercioli A Fabre M , et al. Statin treatment is associated with reduction in serum levels of receptor activator of NF-κB ligand and neutrophil activation in patients with severe carotid stenosis. Mediators Inflamm. 2014;2014:1-720987. 10.1155/2014/720987 PMC3933515 24648660

[15] Vuilleumier N Montecucco F Spinella G , et al. Serum levels of anti-apolipoprotein A-1 auto-antibodies and myeloperoxidase as predictors of major adverse cardiovascular events after carotid endarterectomy. Thromb Haemost. 2013;109:706-715. 23364307 10.1160/TH12-10-0714

[16] Messas E Goudot G Halliday A , et al. Management of carotid stenosis for primary and secondary prevention of stroke: state-of-the-art 2020: a critical review. Eur Heart J Suppl. 2020;22:M35-M42. 10.1093/eurheartj/suaa162 PMC7916422 33664638

[17] Bonati LH Jansen O de Borst GJ Brown MM. Management of atherosclerotic extracranial carotid artery stenosis. Lancet Neurol. 2022;21:273-283. 35182512 10.1016/S1474-4422(21)00359-8

[18] Lanza G Orso M Alba G , et al. Guideline on carotid surgery for stroke prevention: updates from the Italian Society of vascular and endovascular surgery. A trend towards personalized medicine. J Cardiovasc Surg. 2022;63:471-491. 35848869 10.23736/S0021-9509.22.12368-2

[19] Naylor AR Rothwell PM Bell PR. Overview of the principal results and secondary analyses from the European and North American randomised trials of endarterectomy for symptomatic carotid stenosis. Eur J Vasc Endovasc Surg. 2003;26:115-129. 12917824 10.1053/ejvs.2002.1946

[20] Gough MJ ; GALA Trial Collaborators. The GALA Trial–a summary of the findings. Eur J Vasc Endovasc Surg. 2008;36:505-506. 18815058 10.1016/j.ejvs.2008.09.001

[21] Dharmakidari S Bhattacharya P Chaturvedi S. Carotid artery stenosis: medical therapy, surgery, and stenting. Curr Neurol Neurosci Rep. 2017;17:77-7. 10.1007/s11910-017-0786-2 28825185

[22] Lindholt JS Vammen S Juul S Henneberg EW Fasting H. The validity of ultrasonographic scanning as screening method for abdominal aortic aneurysm. Eur J Vasc Endovasc Surg. 1999;17:472-475. 10375481 10.1053/ejvs.1999.0835

[23] Long A Rouet L Lindholt JS Allaire E. Measuring the maximum diameter of native abdominal aortic aneurysms: review and critical analysis. Eur J Vasc Endovasc Surg. 2012;43:515-524. 22336051 10.1016/j.ejvs.2012.01.018

[24] Hansen NJ Kaza RK Maturen KE Liu PS Platt JF. Evaluation of low-dose CT angiography with model-based iterative reconstruction after endovascular aneurysm repair of a thoracic or abdominal aortic aneurysm. AJR Am J Roentgenol. 2014;202:648-655. 24555604 10.2214/AJR.13.11286

[bibr25-11772719251324322] Engellau L Albrechtsson U Dahlström N , et al. Measurements before endovascular repair of abdominal aortic aneurysms. MR imaging with MRA vs. Angiography and CT. Acta Radiol. 2003;44:177-184. 12694105 10.1080/j.1600-0455.2003.00029.x

[bibr26-11772719251324322] Palombo D Morbelli S Spinella G , et al. A positron emission tomography/computed tomography (PET/CT) evaluation of asymptomatic abdominal aortic aneurysms: another point of view. Ann Vasc Surg. 2012;26:491-499. 22197524 10.1016/j.avsg.2011.05.038

[27] Henderson EL Geng YJ Sukhova GK , et al. Death of smooth muscle cells and expression of mediators of apoptosis by T lymphocytes in human abdominal aortic aneurysms. Circulation. 1999;99:96-104. 9884385 10.1161/01.cir.99.1.96

[28] Annaratone L De Palma G Bonizzi G , et al. Basic principles of biobanking: from biological samples to precision medicine for patients. Virchows Arch. 2021;479:233-246. 34255145 10.1007/s00428-021-03151-0 PMC8275637

[29] Zika E Paci D Braun A , et al. A European survey on biobanks: trends and issues. Public Health Genomics. 2011;14:96-103. 20395653 10.1159/000296278

[30] Zhu Y Jackson D Hunter B , et al. Models of cardiovascular surgery biobanking to facilitate translational research and precision medicine. ESC Heart Fail. 2022;9:21-30. 34931483 10.1002/ehf2.13768 PMC8787984

[31] Malsagova K Kopylov A Stepanov A , et al. Biobanks-a platform for scientific and biomedical research. Diagnostics. 2020;10:485. 32708805 10.3390/diagnostics10070485 PMC7400532

[32] Coppola L Cianflone A Grimaldi AM , et al. Biobanking in health care: evolution and future directions. J Transl Med. 2019;17:172. 31118074 10.1186/s12967-019-1922-3 PMC6532145

[33] Iacoviello L De Curtis A Donati MB de Gaetano G. Biobanks for cardiovascular epidemiology and prevention. Future Cardiol. 2014;10:243-254. 24762252 10.2217/fca.13.110

[34] Crea F Libby P. Acute coronary syndromes: the way forward from mechanisms to precision treatment. Circulation. 2017;136:1155-1166. 28923905 10.1161/CIRCULATIONAHA.117.029870 PMC5679086

[35] Montecucco F Lenglet S Gayet-Ageron A , et al. Systemic and intraplaque mediators of inflammation are increased in patients symptomatic for ischemic stroke. Stroke. 2010;41:1394-1404. 20538699 10.1161/STROKEAHA.110.578369

[36] Carbone F Satta N Burger F , et al. Vitamin D receptor is expressed within human carotid plaques and correlates with pro-inflammatory M1 macrophages. Vasc Pharmacol. 2016;85:57-65. 10.1016/j.vph.2016.08.004 27555526

[37] Lederle FA. Screening for AAA in the USA. Scand J Surg. 2008;97:139-141. 18575032 10.1177/145749690809700213

[38] Giardina S Pane B Spinella G , et al. An economic evaluation of an abdominal aortic aneurysm screening program in Italy. J Vasc Surg. 2011;54:938-946. 21820837 10.1016/j.jvs.2011.03.264

[39] De Negri Atanasio G Ferrari PF Campardelli R Perego P Palombo D. Poly(lactic-co-glycolic acid) nanoparticles and nanoliposomes for protein delivery in targeted therapy: a comparative in vitro study. Polymers. 2020;12:2566. 33139610 10.3390/polym12112566 PMC7692461

[40] De Negri Atanasio G Ferrari PF Baião A , et al. Bevacizumab encapsulation into PLGA nanoparticles functionalized with immunouteroglobin-1 as an innovative delivery system for atherosclerosis. Int J Biol Macromol. 2022;221:1618-1630. 35970371 10.1016/j.ijbiomac.2022.08.063

