# Supplementary material for: The Genoa Vascular Biobank: A Today Resource for Future Perspectives in Vascular Research
Source: Biomark Insights. 2025 Jul 13;20:11772719251324322. doi: 10.1177/11772719251324322 (PMC12256751; doi:10.1177/11772719251324322)
Supplement: sj-docx-1-bmi-10.1177_11772719251324322 – Supplemental material for The Genoa Vascular Biobank: A Today Resource for Future Perspectives in Vascular Research [file sj-docx-1-bmi-10.1177_11772719251324322.docx]

**Supplementary Material and Methods**

**Isolation of peripheral blood mononuclear cell (PBMCS) from blood sample:**

- Diluite 6 ml of blood with an equal volume of saline solution in 15 mL tube (tube 1).
- Add 3 ml of Lympholyte separation medium (Cedarlane, Canada) in 15 mL tube (tube 2).
- Gently layer 12 mL of pre-diluted blood into tube 2.
- Centrifuge at 1800 × g for 20 minutes without brake.
- Carefully aspirate and discard the top plasma layer above the interphase of the PBMC ring.
- Transfer it into a new 15 ml tube (tube 3).
- Resuspend cells with 12 ml of normal saline solution
- Centrifuge at 1600 × g for 6 minutes with brake.
- Discard the supernatant and add 2 ml of normal saline solution and divide them into four aliquots (250 μl of volume for each).
- Centrifuge at high-speed for 30 seconds. Discard the supernatant and store at -80
