# Supplementary figures and images for: The Genoa Vascular Biobank: A Today Resource for Future Perspectives in Vascular Research
Source: Biomark Insights. 2025 Jul 13;20:11772719251324322. doi: 10.1177/11772719251324322 (PMC12256751; doi:10.1177/11772719251324322)

## Slide 1
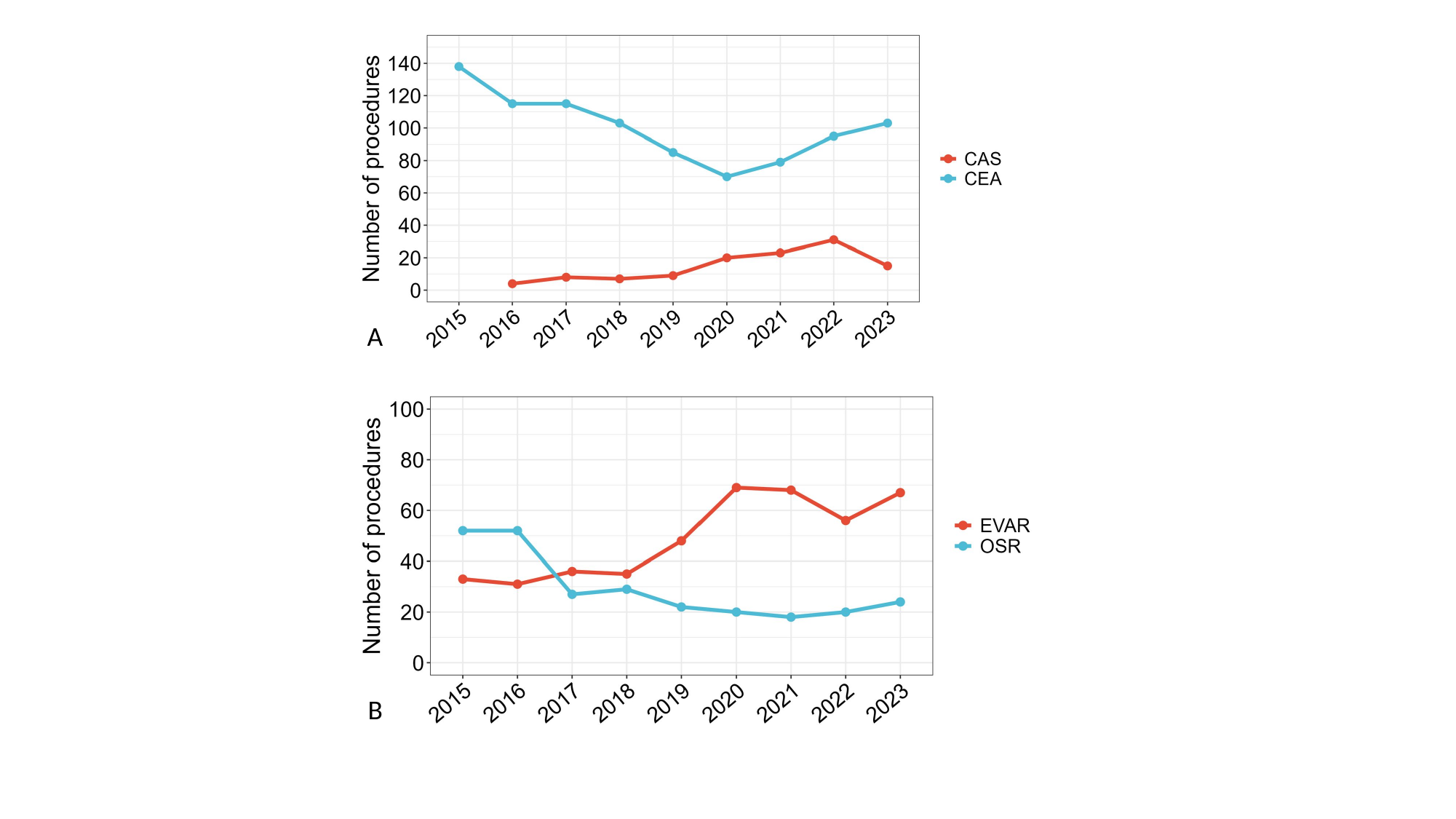

A
B

Supplement: sj-pptx-2-bmi-10.1177_11772719251324322 – Supplemental material for The Genoa Vascular Biobank: A Today Resource for Future Perspectives in Vascular Research [file sj-pptx-2-bmi-10.1177_11772719251324322.pptx]
